# Seroepidemiology of cytomegalovirus infection in pregnant women in Durango City, Mexico

**DOI:** 10.1186/1471-2334-14-484

**Published:** 2014-09-05

**Authors:** Cosme Alvarado-Esquivel, Jesús Hernández-Tinoco, Luis Francisco Sánchez-Anguiano, Agar Ramos-Nevárez, Sandra Margarita Cerrillo-Soto, Sergio Estrada-Martínez, Lucio Martínez-Ramírez, Alma Rosa Pérez-Álamos, Carlos Alberto Guido-Arreola

**Affiliations:** Biomedical Research Laboratory, Faculty of Medicine and Nutrition, Juárez University of Durango State, Avenida Universidad S/N, 34000 Durango, Mexico; Institute for Scientific Research “Dr. Roberto Rivera-Damm”, Juárez University of Durango State, Avenida Universidad S/N, 34000 Durango, Mexico; Clínica de Medicina Familiar, Instituto de Seguridad y Servicios Sociales de los Trabajadores del Estado, Predio Canoas S/N, 34079 Durango, Mexico

**Keywords:** Cross-sectional study, Seroprevalence, Cytomegalovirus, Infection, Epidemiology, Mexico

## Abstract

**Background:**

Cytomegalovirus causes congenital infections all around the world. The seroepidemiology of cytomegalovirus infection in pregnant women in Mexico is largely unknown. We sought to determine the seroprevalence of cytomegalovirus infection in pregnant women in Durango City, Mexico; and to determine seroprevalence association with socio-demographic, clinical and behavioral characteristics of pregnant women.

**Methods:**

Through a cross-sectional study design, 343 pregnant women were examined for anti-cytomegalovirus IgG and IgM antibodies in Durango City, Mexico. We used a standardized questionnaire to obtain the general characteristics of the pregnant women. Multivariate analysis was performed to determine the association of cytomegalovirus infection with the characteristics of the pregnant women.

**Results:**

Anti-CMV IgG and IgM antibodies were detected in 225 (65.6%) and in none of the 343 pregnant women studied, respectively. Multivariate analysis showed that CMV exposure was associated with increasing age (OR = 1.67; 95% CI: 1.01-2.76; *P* = 0.04). Other women characteristics including socioeconomic status, education, blood transfusion, transplantation, sexual promiscuity and number of previous pregnancies or deliveries did not show an association with CMV exposure.

**Conclusions:**

This is the first seroepidemiology study of CMV infection in pregnant women in Mexico. A number of known factors associated with CMV infection were not associated with CMV exposure in the women studied. Further studies to determine routes of CMV infection in pregnant women in Mexico are needed.

## Background

Cytomegalovirus (CMV) is a DNA virus of the Betaherpesvirinae subfamily and Herpesviridae family [1]. CMV is a leading cause of congenital infections all around the world [2–4]. Congenital infections with CMV may be asymptomatic [5] or may lead to hearing impairment, mental retardation, cerebral palsy [6], and neurodevelopmental disabilities [2, 4]. The incidence of congenital CMV infection is about 1%-7% of births [4]. In addition, congenital CMV infection may occur not only in primary but also in non-primary maternal infections [4]. Infections with CMV persist through chronic and latent states of infections [7], and can be reactivated with shedding of infectious virus [1, 8]. Transmission of CMV occurs by person-to-person contact [1]. In addition, CMV infection acquired by blood transfusion may lead to significant complications in immunocompromised individuals [9]. Severe disease including pneumonia, retinitis, hepatitis, encephalitis, and other organ involvements due to CMV in immunocompromised patients have been reported [10].

Very little is known about the seroepidemiology of CMV infection in Mexican populations. We are not aware of any study on the seroepidemiology of CMV infection in pregnant women in Mexico. Therefore, we sought to determine the seroprevalence of CMV infection in pregnant women in Durango City, Mexico. In addition, the socio-demographic, clinical and behavioral characteristics of pregnant women associated with CMV infection were also investigated.

## Methods

### Study population and study design

Through a cross-sectional study design, we studied pregnant women in a public primary health care center (Clínica de Medicina Familiar, Instituto de Seguridad y Servicios Sociales de los Trabajadores del Estado) in Durango City, Mexico from April to November 2013. Inclusion criteria for enrollment in the study were: 1) pregnant women; 2) residing in Durango City; 3) aged 15 years and older; and 4) who accepted to participate in the study.

### Socio-demographic, clinical and behavioral data

We submitted a standardized questionnaire to obtain socio-demographic, clinical and behavioral characteristics from the pregnant women studied. Socio-demographic data included age, birthplace, residence, occupation, and educational and socio-economic status. Clinical data included obstetric history (month of pregnancy, number of pregnancies, deliveries, cesarean sections and miscarriages), presence of any underlying disease, history of lymphadenopathy, frequent headaches and impairments of memory, reflexes, vision and hearing, and history of hepatitis, blood transfusions, transplants or surgery. Behavioral data included addictions, sexual promiscuity, washing hands before eating, eating away of home (in restaurants and fast food outlets), consumption of unpasteurized milk, untreated water, and unwashed raw vegetables or fruits, foreign travel, and type of flooring at home.

### Laboratory tests

Serum samples were obtained from each pregnant women by centrifugation of whole blood. Sera of the women were kept frozen until analyzed. Sera were examined for anti-CMV IgG antibodies by a commercially available enzyme immunoassay “Cytomegalovirus IgG (CMV IgG)” kit (Diagnostic Automation Inc., Calabasas, CA, USA) and for anti-CMV IgM antibodies by a commercially available enzyme immunoassay “Cytomegalovirus IgM (CMV IgM” kit (Diagnostic Automation Inc., Calabasas, CA, USA). The tests were performed following the instructions of the manufacturer. The cut-off values for IgG and IgM seropositivity were obtained as follows: firstly, the mean optical densities of IgG and IgM calibrators were multiplied by the correction factor (0.50-0.55) printed on the label of calibrators to obtained the corrected mean cut-off value: then, we calculated the CMV G and M indexes by dividing the optical density of each sample by the corrected mean cut-off value. A sample was considered positive for IgG or IgM when a CMV G index or a CMV M index was greater than 1.1, respectively. These assays are qualitative, and no quantification of antibody levels were performed.

### Ethical aspects

This study was approved by the ethical committee of the Instituto de Seguridad y Servicios Sociales de los Trabajadores del Estado in Durango City. The purpose and procedures of the study were explained to all pregnant women, and a written informed consent was obtained from all of them and from the next of kin of minor participants.

### Statistical analysis

The statistical analysis was performed with the aid of the software: Epi Info version 7 and SPSS version 15.0. For calculation of the sample size, we used a value of 15,000 as a population size from which the sample was selected, a reference seroprevalence of 89.2% [11] as expected frequency of the factor under study, 3.5% of confidence limits, a design effect of 1.0, one cluster, and a confidence level of 95%. The result of the calculation was 296 subjects. We used the Pearson’s chi square and the Fisher exact test (when values were small) for comparison of the frequencies among groups. Bivariate and multivariate analyses were used to evaluate the association between the characteristics of the women and CMV seropositivity. Variables with a *P* value equal to or less than 0.10 obtained in the bivariate analysis were included in the multivariate analysis. Odd ratios (OR) and 95% confidence intervals (CI) were calculated by multivariate analysis using the Enter method. A *P* value less than 0.05 was considered statistically significant.

## Results

In total, we enrolled 343 pregnant women. Most of them were born in Durango; their mean age was 28.88 ± 6.12 years (range 15–43 years). General socio-demographic and obstetric characteristics of the pregnant women studied are shown in Table 1. Anti-CMV IgG antibodies were detected in 225 (65.6%) of the 343 pregnant women studied. None of the 343 pregnant women had anti-CMV IgM antibodies.

**Table 1:** **Socio**-**demographic and obstetric characteristics of pregnant women and seroprevalence of CMV infection**

	No. of women	Prevalence of CMV infection		P value
Characteristic	tested^a^	No.	%	
Age groups (years)				
15-30	198	119	60.1	0.009
31-43	144	106	73.6	
Birth place				
Durango State	319	209	65.5	0.68
Other Mexican State	20	14	70.0	
Residence place				
Durango State	342	224	65.5	1.00
Other Mexican State	1	1	100.0	
Residence area				
Urban	325	213	65.5	0.75
Suburban	5	4	80.0	
Rural	13	8	61.5	
Educational level				
Up to 6 years	1	1	100.0	0.75
7-12 years	133	88	66.2	
13 or more years	209	136	65.1	
Occupation				
Unemployed^b^	110	69	62.7	0.44
Employed^c^	233	156	67.0	
Socio-economic level				
Low	19	14	73.7	0.69
Medium	316	206	62.5	
High	4	3	75.0	
Month of pregnancy				
1 to 3	117	77	65.8	0.91
4 to 6	153	100	65.4	
7 to 9	66	45	68.2	
Deliveries				
Yes	140	94	67.1	0.59
No	202	130	64.4	
Pregnancies				
3 or more	228	158	69.3	0.04
1 or 2	115	67	58.3	
Cesarean sections				
None	241	155	64.3	0.47
1 or 2	101	69	68.3	
Miscarriages				
None	269	173	64.7	0.37
1-3	73	51	69.9	

^a^Pregnant women with available data.

^b^Unemployed = none occupation, student or housewife.

^c^Employed = Employee, professional, business, or other.

Of the socio-demographic characteristics of the pregnant women (Table 1), only the variable age showed a *P* value <0.10 by bivariate analysis. Other socio-demographic characteristics including birthplace, residence, educational level, occupation and socio-economic status had *P* values >0.10 by bivariate analysis.

With respect to behavioral characteristics, smoking was the only variable that showed a *P* value <0.10 by bivariate analysis. Other behavioral characteristics including foreign travel, eating away of home, consumption of untreated water, and unwashed raw vegetables or fruits, drug or alcohol addictions, sexual promiscuity, washing hands before eating, and type of flooring at home show *P* values >0.10 by bivariate analysis.

Concerning clinical characteristics, seroprevalence of CMV infection was similar in ill (7/15: 46.7%) than in healthy (217/327: 66.4%) pregnant women (*P* = 0.11). In contrast, seroprevalence of CMV infection was significantly higher (*P* = 0.04) in pregnant women with history of lymphadenopathy (39/50: 78.0%) than in those without such history (186/292: 63.7%). Pregnant women with more than two pregnancies had a significantly higher (*P* = 0.04) seroprevalence of CMV exposure (158/228: 69.3%) than those with one or two pregnancies (67/115: 58.3%). Other clinical characteristics of pregnant women including impairments of memory, reflexes, vision and hearing, history of hepatitis, blood transfusions, transplants or surgery, and other obstetric characteristics (month of pregnancy, number of deliveries, cesarean sections and miscarriages) were not associated with CMV infection.

Multivariate analysis of socio-demographic, clinical and behavioral variables with *P* values equal to or lower than 0.10 by bivariate analysis (Table 2) showed that CMV infection was positively associated only with age (OR = 1.67; 95% CI: 1.01-2.76; *P* = 0.04).

**Table 2:** **Multivariate analysis of selected characteristics of pregnant women and their association with CMV infection**

Characteristic	Odds ratio^a^	95%confidence interval	*P*value
Age (31–43 years)	1.67	1.01 - 2.76	0.04
Lymphadenopathy	2.03	0.98 - 4.19	0.05
More than 2 pregnancies	1.36	0.81 - 2.26	0.23
Smoking	0.53	0.26 - 1.07	0.08

^a^Adjusted by the characteristics included in this Table.

## Discussion

There is a lack of knowledge about the epidemiology of CMV infection in pregnant women in Mexico. The present study was performed to investigate the seroprevalence and correlates of CMV infection in pregnant women in the northern Mexican city of Durango. We found a 65.6% seroprevalence of CMV infection in the pregnant women studied. Based on the seropositivity to CMV IgG and IgM antibodies, all women with CMV exposure had latent infection and none had recent or acute CMV infection. We are not aware of previous reports about the seroepidemiology of CMV infection in pregnant women in Mexico. Therefore, we cannot compare our seroprevalence results with others in pregnant women in Mexico. In a study in healthy women of reproductive age in Cuernavaca City in central Mexico, researchers found a 92.6% seroprevalence of CMV infection [12]. While in a national survey in subjects aged 1 to 70 years old in Mexico an 88.2% seroprevalence of CMV infection was found [11]. On the other hand, comparison of our seroprevalence with those reported in other countries suggests that the seroprevalence of CMV infection in pregnant women in Durango could be placed in an intermediate position of endemicity. The 65.6% seroprevalence found in pregnant women in Durango is similar to the 62.4%-66% seroprevalences reported in pregnant women in Poland [13], Japan [14], and Norway [15], and higher than the 49% seroprevalence in white British pregnant women in the United Kingdom [16] and a 42.3% seroprevalence in pregnant women in Germany [17]. In contrast, the seroprevalence found in pregnant women in Durango is lower than the 92.6%-100% seroprevalences reported in pregnant women in Iran [18], Palestine [19], Brazil [20], Turkey [21], Nigeria [22], and Cuba [23].

Analysis of the socio-demographic characteristics showed that the seroprevalence of CMV infection in pregnant women increased with age. This finding is consistent with those reported in other studies where researchers found an increase in the seroprevalence of CMV infection with age in both pregnant women [13] and general population [24]. Other known contributing factors for CMV infection including socioeconomic status [8], education, sexual promiscuity [12], blood transfusion and transplantation [25] did not show any association with CMV exposure in the women studied. Of the sources of infection with CMV in pregnant women stands out young children [26, 27]. We assess the contact with young children by evaluating the number of children the women have already born (history of pregnancies), considering that women with more than two pregnancies had a higher contact with children than women with fewer pregnancies. Analysis showed that women with more than two pregnancies had a higher, but not statistically significant, seroprevalence of CMV infection than those with one or two pregnancies. This approach was a limitation of the study since contact with children can occur not only at home but also at work in some women. In the present study, the occupation of housewife was not associated with CMV exposure. None of the pregnant women studied reported to be schoolteacher or baby-sitter and the lack of these occupations among the studied women might have a negative influence on the seroprevalence. Therefore, the association of CMV exposure with contact with children should be further evaluated in pregnant women in Mexico. Hygiene practices are important in the prevention of CMV infection [26]. However, in this study the variable washing hands before eating did not show an association with CMV exposure.

## Conclusions

This is the first seroepidemiology study of CMV infection in pregnant women in Mexico. A number of known factors associated with CMV infection were not associated with CMV exposure in the women studied. Further studies to determine routes of CMV infection in pregnant women in Mexico are needed.
